# A simple prediction tool for inhaled corticosteroid response in asthmatic children

**DOI:** 10.1186/s12890-017-0533-0

**Published:** 2017-12-07

**Authors:** Yi-Fan Wu, Ming-Wei Su, Bor-Luen Chiang, Yao-Hsu Yang, Ching-Hui Tsai, Yungling L. Lee

**Affiliations:** 1 Department of Family Medicine, Taipei City Hospital, Renai Branch, Taipei, Taiwan; 2 Institute of Epidemiology and Preventive Medicine, College of Public Health, National Taiwan University, Taipei, Taiwan; 3 Department of Psychology, National Chengchi University, Taipei, Taiwan; 4 Institute of Biomedical Sciences, Academia Sinica, Taipei, Taiwan; 5 Department of Pediatrics, National Taiwan University Hospital, Taipei, Taiwan

**Keywords:** Childhood asthma, Inhaled corticosteroid, Drug response, Prediction model

## Abstract

**Background:**

Inhaled corticosteroids are recommended as the first-line controller medication for childhood asthma owing to their multiple clinical benefits. However, heterogeneity in the response towards these drugs remains a significant clinical problem.

**Methods:**

Children aged 5 to 18 years with mild to moderate persistent asthma were recruited into the Taiwanese Consortium of Childhood Asthma Study. Their responses to inhaled corticosteroids were assessed based on their improvements in the asthma control test and peak expiratory flow. The predictors of responsiveness were demographic and clinical features that were available in primary care settings. We have developed a prediction model using logistic regression and have simplified it to formulate a practical tool. We assessed its predictive performance using the area under the receiver operating characteristic curve.

**Results:**

Of the 73 asthmatic children with baseline and follow-up outcome measurements for inhaled corticosteroids treatment, 24 (33%) were defined as non-responders. The tool we have developed consisted of three predictors yielding a total score between 0 and 5, which are comprised of the following parameters: the age at physician-diagnosis of asthma, sex, and exhaled nitric oxide. Sensitivity and specificity of the tool for prediction of inhaled corticosteroids non-responsiveness, for a score of 3, were 0.75 and 0.69, respectively. The areas under the receiver operating characteristic curve for the prediction tool was 0.763.

**Conclusions:**

Our prediction tool represents a simple and low-cost method for predicting the response of inhaled corticosteroids treatment in asthmatic children.

## Background

Asthma is a chronic complex airway disease characterized by reversible airflow obstruction, bronchial hyper-reactivity, and underlying inflammation. It affects approximately 300 million people worldwide [1] and is one of the most common chronic childhood conditions. Epidemiologic data show that the prevalence of the disease increased by 4.6% per year from 1980 to 1996 in US children and 9.1% of US children (6.7 million) had asthma in 2007 [2]. In Taiwan, the prevalence of asthma in children is around 7.5–20% [3, 4].

The three major classes of controller medication for asthma include inhaled corticosteroids (ICS), β2-agonists, and leukotriene antagonists. However, there is significant variability in the response to these medications [5, 6]. Although ICS is currently recommended as the first-line therapy by the Global Initiative for Asthma, the significant heterogeneity in its efficacy is evident with there being as much as 22–60% of non-responders in those asthmatic children and adults treated with ICS [6–8].

Many clinical features are reported to be associated with ICS efficacy in asthmatic children. Szefler et al. found that higher exhaled nitric oxide (eNO), blood eosinophil counts and serum immunoglobulin E (IgE) were associated with a better forced expiratory volume in 1 s (FEV_1_) response to ICS [7]. Knuffman et al. used the percentage of asthma control days to evaluate response to ICS and found that parental asthma could be another predictor [9]. Furthermore, female sex, [10] higher bronchodilator response, [10] and normal body weight [11] have also been shown to be associated with a favorable response to ICS. Despite these observations, no prior study has provided clinicians with an efficient method to predict ICS response. The purpose of our study is to establish a clinical tool for predicting which asthmatic children are suitable for ICS treatment, as a way to progress towards personalized therapy.

## Methods

### Study population

Taiwanese Consortium of Childhood Asthma Study (TCCAS) was a consortium-based study for childhood asthma. We coordinated with several pediatric asthma specialty clinics in Taiwan. Recruitment of asthmatic children between 5 and 18 years of age started in May 2013 and is still ongoing. Asthma status was determined by asthma specialists, and the age of onset was below 10 years. The exclusion criteria were children with cancer and major immunological diseases, such as systemic lupus erythematosus, rare hereditary diseases, or severe infection. Throughout the study period, each study participant was evaluated by pediatric asthma specialists based on “the classification of asthma severity” and “the level of asthma control” according to the Global Initiative for Asthma guideline [12]. The study protocol has been approved by the National Taiwan University Hospital Research Ethics Committee.

During the study, a life chart with clinical outcome measurements, such as asthma control test (ACT), peak expiratory flow (PEF) and subjective symptoms, was prepared for each asthmatic patient. This life chart also included the dosage and duration of all asthma medications. New users of a controller medication were defined as those who did not use the same prescription for at least four weeks. To ensure the responses to ICS were accurate, only participants with an unstable control status (confirmed by an ACT score < 20 points or a PEF < 90% of the predicted value) at the time of starting a newly prescribed medications and available response records were included for drug response analysis. Subjects who received two first line controller medications in the beginning were excluded.

### Choice of potential predictive factors

Each participant’s parent or guardian was asked to answer a detailed basic characteristics questionnaire, which included age, sex, parental educational level, family income, family history of atopic diseases, body mass index, age at diagnosis, and so on. Information on environmental factors (pet ownership, home dampness, incense burning, and tobacco smoke exposure) as well as prenatal and postnatal information (maternal smoking during pregnancy, gestational age, low birth weight, breast-feeding, and neonatal special care) were also collected.

Subjects underwent approved eNO measurements using the NIOX® Nitric Oxide Monitoring System and the NIOX MINO® Airway Inflammation Monitor [13]. Peripheral blood samples were collected during enrollment for routine blood tests and measurement of the plasma IgE levels.

### Therapeutic response definition

We first measured the baseline ACT and PEF at the time a new asthma controller medication was prescribed. Follow-up ACT and PEF were collected 4 to12 weeks later. The Chinese version of the Childhood Asthma Control Test [14, 15] is a 7-item questionnaire that assesses interference with activity, shortness of breath, nocturnal symptoms, rescue medication use and self-rating of asthma control. The responders were defined as those who improved their post-treatment ACT from <20 points at baseline to ≥20 points or those with ACT ≥20 points at baseline with a 10% improvement in PEF. Subjects, who did not meet the above criteria or started to use other controller medication during follow-up period, were categorized as non-responders.

### Prediction model development

We reviewed the literature to identify relevant risk factors for not responding to ICS in childhood asthma. Our priority lies in the development of a simple, efficient tool for identifying non-responders to ICS. As such, we selected risk factors that could easily be obtained in primary care settings and would not require repeated measurements. We used logistic regression to develop the prediction model when the Chi-Square or t-test *p*-values were lower than 0.15 between ICS responders and non-responders. At last, only the variables with p-value lower than 0.05 in multi-variate analysis were selected to be included in the final prediction tool.

To assess overall performance, we calculated the scaled Brier score, [16] which measures the discrepancy between the predicted probability and the actual outcome. A scaled Brier score of zero means that the model does not predict ICS non-responsiveness in a subject better than the value obtained from the average prevalence of ICS non-responders in childhood asthma; whereas, the maximum value of 1 indicates perfect prediction. To determine the ability of the model to distinguish between ICS responders and non-responders, we plotted the receiver operating characteristic curve and calculated the area under the curve (AUC), which is also known as the c-statistic [16, 17]. The AUC can range from 0 to 1, with 1 being a perfectly discriminating model. Discrimination is considered not better than chance if the AUC is 0.5, moderate if the AUC is 0.6 to 0.8, and good if the AUC is greater than 0.8 [17]. The calibration of the model was tested by using the Hosmer-Lemeshow goodness-of-fit test [18]. A Hosmer-Lemeshow goodness-of-fit test result <0.05 indicates that the predicted probabilities and the actual outcome are in poor agreement.

### Clinical prediction tool

To make our model easier to apply, we shifted the continuous variables to binary predictors based on the cut-off point established by a literature review. The predictors were then weighted by rounding their regression coefficients to the nearest integer. Finally, we used a scoring system to build a clinical prediction tool and attempted to maintain a comparable predictive function.

### Sample size calculation

In a prior study, Cowan DC et al. used the data from 46 asthmatic patients and found that the odds ratio of having a steroid response versus having no steroid response for high eNO value (>35 ppm) was 6.0. [19] In addition, we assumed the prevalence of ICS non-responders was 40% (22–60% from literature review) and that low eNO prevalence was 0.63 [20]. Thus, for one variable of odds ratio that equals to 6.0, a sample size of 51 total patients would be needed to provide 80% power with a 5% two-sided type I error.

## Results

Of the 158 subjects in the current TCCAS database, 105 matched the inclusion criteria for drug response analysis. Eight subjects were excluded from analysis because they were initiated on leukotriene antagonist and ICS in the beginning. Of the remaining 97 subjects, 73 were defined as new ICS users. Based on our defined outcome, approximately 33% of the new ICS users were non-responders (24 cases).

Basic characteristics and relevant risk factors evaluated in our TCCAS are listed in Table 1. We used logistic regression to build the main prediction model with the following three variables: the age at diagnosis, gender, and eNO (Table 2). The overall performance, measured by the scaled Brier score, was 0.23 and its discriminative ability (AUC) was 0.763. The calibration by Hosmer- Lemeshow test showed fair agreement between the predicted probabilities of no response to ICS and the observed frequencies in asthmatic children (*p* = 0.26).

**Table 1:** Basic characteristics of inhaled corticosteroid users in TCCAS

	Non-responders (*n* = 24)	Responders (*n* = 49)	*p* value ^a^
Age	9.8 (3.3)	10.5 (3.5)	0.47
Female	6 (25.0)	25 (51.0)	0.03
Age at physician-diagnosis	5.0 (2.6)	3.7 (1.6)	0.03
Second-hand smoke exposure	8 (33.0)	15 (30.6)	0.81
Parental asthma	6 (25.0)	10 (20.4)	0.66
Overweight	10 (41.7)	13 (26.5)	0.19
Immunoglobin E (IU/ml)	574.5 (528.4)	649.9 (600.1)	0.60
Blood neutrophil (%)	49.1 (13.8)	47.0 (12.1)	0.53
Blood eosinophil (%)	5.1 (3.9)	5.7 (3.6)	0.51
Exhaled nitric oxide (ppb)	24.7 (33.1)	36.1 (31.0)	0.15

Numbers are present as mean (SD) or *n* (%)

^a^Calculated by t-test or chi-square test

**Table 2:** Coefficients, odds ratio, and 95% confidence intervals (CI) for important factors to predict ICS non-responders in asthmatic children

	Unadjusted continue variable			Unadjusted binary variable ^a^			Adjusted binary variable ^a, b^		
	ß	OR	95% CI	ß	OR	95% CI	ß	OR	95% CI
Male sex	–	–	–	1.14	3.13	0.98–9.94	1.52	4.59	1.36–15.48
Age at physician-diagnosis	0.33	1.39	1.07–1.80	1.20	3.33	1.00–11.14	1.48	4.44	1.11–17.77
Exhaled nitric oxide	−0.01	0.99	0.97–1.01	1.27	3.57	1.06–12.06	1.66	5.25	1.34–20.60

^a^Binary variables were defined as the age of physician-diagnosis late than 5 years old, exhaled nitric oxide less or equal to 35 ppb

^b^The model included male sex, age at physician-diagnosis and exhaled nitric oxide

As the majority of patients with childhood asthma presenting before 5 years of age showed strong familial aggregation [21] and parental asthma is an indicator of response to ICS, [9] we determined that the age at physician-diagnosis later than 5 years old was a predictor of failed response to ICS. In addition, according to the American Thoracic Society documents, [22] eNO greater than 35 ppb in children could indicate eosinophilic inflammation and sensitivity to corticosteroids.

**Fig. 1:**
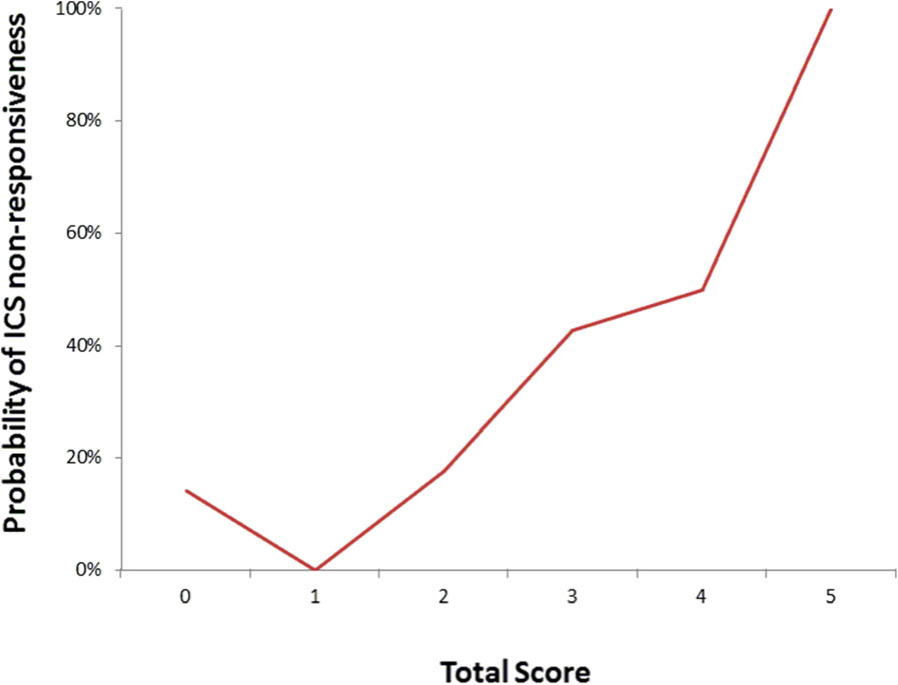
The estimated probability of ICS non-responsiveness against different total scores

**Fig. 2:**
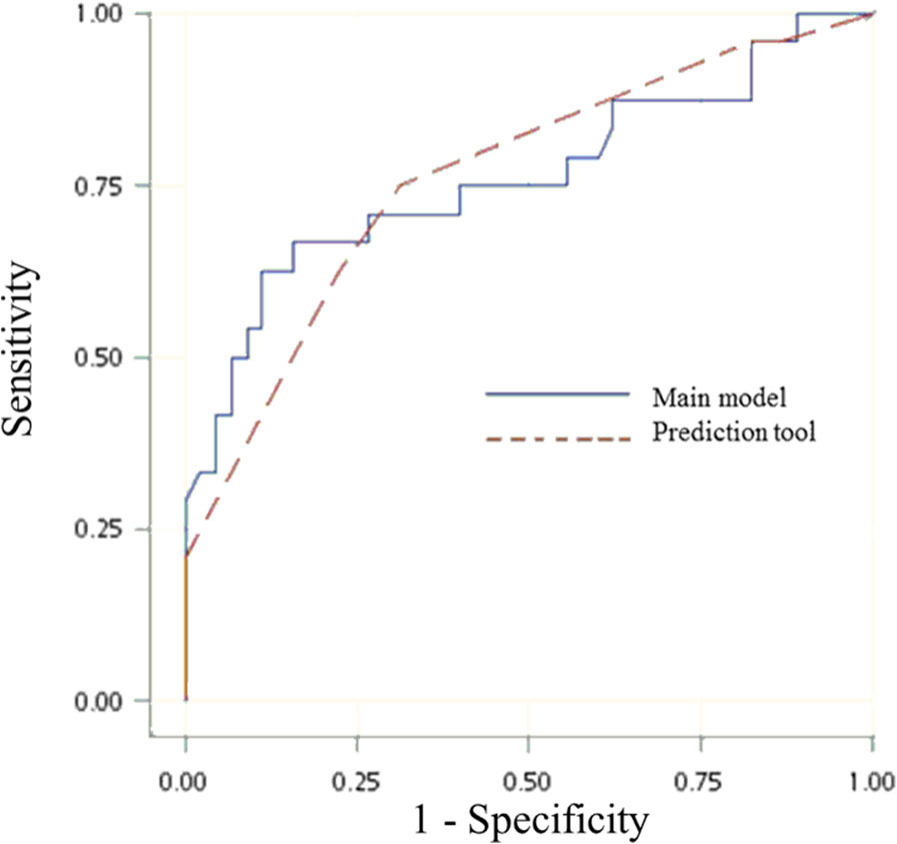
Receiver operating characteristic curve for the main model and clinical prediction tool

Multivariate logistic regression analysis with these binary predictors was also performed (Table 2). However, given the limited numbers of non-responders, we simplified the prediction model by using a scoring system and avoided having more than two variables in the logistic regression analysis. The final prediction tool included three parameters, and each of them contributed to the prediction score (range from 0 to 5, Table 3 & Fig. 1) with one of three values (0, 1, or 2). It showed similar discriminative ability (AUC = 0.763) when it was compared to the main model (AUC = 0.763) (Fig. 2).

**Table 3:** Parameters in the clinical prediction tool for ICS non-responders

Parameter		Score
Gender	Male	2
	Female	0
Age at physician-diagnosis	> 5 years old	1
	≦5 years old	0
Exhaled nitric oxide	> 35 ppb	0
	≦35 ppb	2
Total score (lowest-highest)		0–5

The sensitivity and specificity of this prediction tool for ICS non-responders was 0.75 and 0.69 (Youden index: 0.44), respectively, when the cut-off score was greater than or equal to 3 (Table 4). We could use this score to define three clinical groups for ICS responsiveness. In our study, the percentage of ICS non-responders in children with scores less than or equal to 2 was 16%, compared to 43% (score equal to 3) and 60% (score greater than or equal to 4) in other groups.

**Table 4:** Performance measures of the prediction tool at different cutoff values

Score cutoff	Sensitivity	Specificity	PPV ^b^	NPV ^b^	LR+	LR-
≧1	0.96	0.13	0.37	0.86	1.10	0.31
≧2	0.96	0.18	0.38	0.89	1.16	0.23
≧3	0.75	0.69	0.56	0.84	2.41	0.36
≧4	0.63	0.78	0.60	0.80	2.81	0.48
5	0.21	1.00	1.00	0.70	^a^	0.79

*LR +* positive likelihood ratio, *LR -* negative likelihood ratio, *NPV* negative predictive value, *PPV* positive predictive value

^a^Great uncertainty of estimate because of sensitivity and specificity close to 0 or 1

^b^Positive and negative predictive value will change depending on the prevalence on non-responders in different populations

## Discussion

Based on the results of our study, we developed our tool for the prediction of ICS response to include the following three parameters: male sex, eNO level of 35 ppb and the physician-diagnosis age of 5 years old. Sensitivity and specificity of the tool for predicting inhaled corticosteroids non-responsiveness, for a score of 3, were 0.75 and 0.69, respectively. Based on our study, we can conclude that it may be suitable to prescribe ICS for children with a score less than or equal to 2 since the non-responders rate was relative low. ICS could be tried with close follow-ups for those children with scores equal to 3 and we may consider other controller medication for children with higher scores. As shown, our tool can be easily applied by primary care physicians.

Asthma is a complex airway disease and there is not one universally accepted indicator, similar to HbA1c for diabetes, to evaluate therapeutic efficacy. In the current guideline for asthma treatment, therapeutic strategy is adjusted on the basis of symptoms, lung function, and acute exacerbations. However, the relationship between these key components of the disease may vary among different asthmatic patients. Compared to previous studies, [7, 10] we used ACT and PEF to define the response of ICS in asthmatic children, which comprehensively included subjective and objective evaluation of improvement after treatment with controller medications. Visits to the emergency department were excluded from our outcome definition due to the low incidence rate (< 3%). This may be attributed to the high accessibility of primary care physicians in Taiwan and the fact that most of the cases in our cohort were classified as mild to moderate severity.

Severe clinical features have been reported to be associated with ICS sensitivity, especially markers of allergic inflammation, such as IgE, blood eosinophil count, and eNO [7, 9, 23]. ICS responders seem to have asthma that is preferentially modulated by the T_H_2 immune pathway [24]. This may be one of the asthma phenotypes that is more prevalent among children than in adults and is associated with atopy, eosinophilic inflammation, and type 1 hypersensitivity reactions.

With respect to the influence of sex on ICS response, the findings of previous studies were inconsistent. Galant et al. found that the female sex was associated with a higher likelihood of responding to ICS therapy, defined as greater than 7.5% increase in FEV_1_ from baseline [10]. On the contrary, in the TREXA trial, male sex was reported to be associated with greater duration of asthma control as a result of daily treatment with ICS [23]. This could be partially explained by differences in ethnicity; therefore, our prediction tool must be carefully generalized when applied to other populations.

Obesity was another factor known to increase the risk of incident asthma [25]; additionally, it influenced the response to ICS [26]. In our study, the proportion of overweight children in the ICS non-responders group was approximately 1.5 fold greater than that in the ICS responders group, and this result is consistent with that of previous studies.

The age of asthma onset has been found to be a determinant of different asthma phenotypes in adults. The age of twelve years was most commonly used as the delineation between two age-of-onset phenotypes. Adults with early-onset current asthma tend to be atopic and have a higher frequency of acute exacerbations, whereas adults with late-onset disease are more likely to be female or smokers [27]. In children, the Severe Asthma Research Program also found that late-onset subjects had relatively normal lung function and less atopy [28]. Using cluster analysis, Chang et al. found one cluster with an average age of onset of 5.9 years, which was different from three other clusters (age of onset: 2.2, 1.4, and 1.8 years). However, the treatment response of ICS in three early-onset clusters was inconsistent [29]. In our study, we first defined the age of 5 years as the cut-off point for differentiating the ICS sensitivity; the odds ratio for being an ICS non-responder for subjects older than 5 years was 4.44 (*p* = 0.04; see Table 2).

Our study had several limitations. Compared to clinical trials, the compliance of the ICS treatment may have been inconsistent in our study subjects, which could affect the accuracy of our evaluation. This type of bias was difficult to avoid in an observational study. We tried to reduce these types of confounders by only enrolling patients who have returned for regular clinic visits. However, despite the possible discrepancy, our observational design could reflect the real efficacy in actual practice settings.

It is reported that certain baseline lung function indices present prior to ICS therapies, such as FEV_1_, [7, 10] bronchodilator response [10] and a provocative dose of methacholine that resulted in a 20% decline in FEV_1_ value [7, 9] are associated with the responsiveness. However, this information was not available for our study subjects. Adding other lung function indices to our prediction tool could improve its performance; however, it could also lose practicality, since younger children may not have qualified lung function results.

Although the ability to discriminate responders from non-responders (denoted by the AUC) was good, there was still one non-responder with no predictive factors to explain the poor response to ICS (Fig. 1). Like other chronic inflammatory disease, genetic issue may result in disease heterogeneity [30, 31]. Further research of possible genetic factors may be helpful for identifying these exceptional cases; nevertheless, cost effectiveness should be taken into consideration.

Finally, there were no independent samples for external validation of this study; therefore, in order to complete the evaluation of this predictive tool, a validation study must be carried out. Currently, there is no practical method for identifying ICS non-responders in clinical practice, therefore we used our cohort as a pilot study to develop the tool and hope it provided a basis for ongoing epidemiologic researches and improvement of clinical primary care.

## Conclusions

Our prediction tool represents a simple and low-cost method for predicting the response of asthmatic children to ICS, and it is ready to be validated with other independent sample groups. We hope our tool can aid clinicians in evaluating the suitability of carrying out ICS treatment in certain patients and facilitate the formulation of customized patient therapy.

## Abbreviations

ACT: Asthma control test
AUC: Area under the curve
eNO: Exhaled nitric oxide
FEV1: Forced expiratory volume in 1 s
ICS: Inhaled corticosteroid
IgE: Immunoglobulin E
PEF: Peak expiratory flow
TCCAS: Taiwanese Consortium of Childhood Asthma Study
